# Supplementary material for: Impact of COVID-19 on clinical practice, income, health and lifestyle behavior of Brazilian urologists
Source: Int Braz J Urol. 2020 Sep 2;46(6):1042–71. doi: 10.1590/S1677-5538.IBJU.2020.99.15 (PMC7527096; doi:10.1590/S1677-5538.IBJU.2020.99.15)
Supplement: Supplementary file 1 [file 1677-6119-ibju-46-06-1042-suppl01.pdf]

APPENDIX - SUPPLEMENTARY MATERIALS

Supplementary material 1 - Web-based survey with 39 questions, translated to English.

1. Impact on urological medical practice

* 1. **Free and informed consent form**

You are being invited to voluntarily participate in a research developed by Brazilian Society of Urology (BSU). After reading this Consent Form, if you agree to participate, click ACCEPT at the end of this document. In case you decide not to participate, click DO NOT ACCEPT.

This is a study to understand in which way COVID-19 has been affected Brazilian urologists. The filling of this question form takes about 10 minutes.

This research will help understand the professional impact of COVID 10 on Brazilian urologists and will help BSU to develop actions to reestablish and refresh urological practice after the end of the COVID-19 pandemic.

Your participation is voluntary and secrecy if guaranteed, it is not necessary to identify yourself. Gathered data will be used in future book, paper or digital communication. The research will not harm you in any way or will generate any financial return. If you have any doubt, please contact the authors by the email: sbuonline@sbunet.org.br.

CONSENT FORM TO PARTICIPATE

I was correctly informed and enlightened by the authors about the research, involved proceedings as well as the possible risks and benefits of my participation.

- ◯ I ACCEPT to participate
- ◯ I DO NOT ACCEPT to participate

2. Knowing the Urologist

* 2. What is your age?

20 50 100

* 3. In which Brazilian state do you currently work?

* 4. Please, inform which alternative better describes your marital/familial status:

- I am married (or have a steady partner) and I have kid (s)
- I am married (or have a steady partner) and don't have kids
- I am single (or divorced/separated/widow) without a steady partner and I have kid(s)
- I am single (or divorced/separated/widow) without a steady partner and I don't have kid(s)
- Another (specify):

* 5. I am

- Male
- Female
- Another (specify)

* 6. Which of the following categories better describes your current work and income in medicine?

- Most of my income as a doctor comes from a public job as urologist
- Most of my income as a doctor comes from a private or no-profit organization job as urologist
- Most of my income as a doctor comes from my private office as urologist
- Most of my income comes from other medical activities than urology (clinical or general practice, etc)
- Most of my income comes from activities other than medicine
- Retired

SOCIEDADE BRASILEIRA DE UROLOGIA

* 7. In the last 4 weeks (COVID-19 epidemics) how many daily hours (of working days) have you been involved in medical practice activities (medical consultations, surgeries, telemedicine, medical reports, on call activities, bureaucratic office work)?

- ◯ I have not practiced medicine in the past weeks
- ◯ Up to 2 hours per day
- ◯ From 2 to 4 hours per day
- ◯ From 4 to 8 hours per day
- ◯ More than 8 hours per day

* 8. In relation to the last 3 months (mean) before COVID-19 epidemics, how many scheduled medical urological consultations have you attended in the last 4 weeks (since the beginning of the epidemics):

- ◯ Remained stable
- ◯ Reduced up to 25%
- ◯ Reduced 25-50%
- ◯ Reduced 50-75%
- ◯ Reduced more than 75%
- ◯ Increased more than 10%
- ◯ Other (specify)

* 9. In relation to the number of elective urologic surgeries (mean) before COVID-19 pandemic, what have you observed in relation to the number of them in the last 4 weeks (since the beginning of the COVID 19 pandemic):

- ◯ Remained stable
- ◯ Reduced up to 25%
- ◯ Reduced 25-50%
- ◯ Reduced 50-75%
- ◯ Reduced more than 75%
- ◯ Increased more than 10%
- ◯ Other (specify)

* 10. In relation to the mean number of urgent urologic surgeries in the past 3 months, what have you observed in relation to them in the past 4 weeks since the beginning of the pandemic:

- Remained stable   Reduced
- up to 25%
- Reduced 25-50%
- Reduced 50-75%
- Reduced more than 75%
- Increased more than 10%
- Other (specify)

3.Professional History

* 11. In the last 4 weeks, how many surgeries have you performed in each of the following priority classification categories:

Emergency: ureteral lithiasis with refractory pain or alteration of renal function or infection or single kidney; ureteral obstruction in pregnant woman; urological abscess; torsion; clot retention; urological bleeding; cadaver donor transplantation; penile fracture; prothesis infection; urological trauma

Priority 1: radical cystectomy; nephrectomy (with thrombus); vesical tumor resection; second time neuromodulator; orchiectomy (tumor); nephroureterectomy; penile cancer; cancer adrenalectomy; urethral stenosis and impending retention.

Priority 2: retroperitoneal lymph node dissection; radical nephrectomy of T3-4 tumors; adrenalectomy (functioning tumor); urinary fistula; asymptomatic ureteral calculus after failed conservative treatment; prostate biopsy in suspected patient with symptoms of advanced disease.

Priority 3: prostatectomy in high risk cancer; partial nephrectomy in tumors T1b-2; replacement of double J stent; sling incision; mash removal; urinary lithiasis in patient with double J stent; retention due to BPH.

Non-essentials: prostatectomy due to low or medium risk tumors; partial nephrectomy in T1a tumors; postectomy; scrotal surgeries; BPH or urethral stenosis without retention; slings prolapse; injection of botulinic toxin; penile prothesis; Peyronie’s disease; live donor transplantation; pediatric genital surgeries; adrenalectomy (no cancer and not functioning); renal lithiasis (**if there were no surgeries in each of the previous categories please mark 0**).

Emergency ______

Priority 1 ______

Priority 2 ______

Priority 3 ______

Non-essentials ______

* 12. Taking into account the previous classification of surgical priorities (previous question), how long do you think it is safe to postpone **Priority 1** surgeries without causing harm to patient (make a general estimate of surgeries of this class of priority)

- ○ Up to 1 month
- ○ Up to 2 months
- ○ Up to 3 months
- ○ Up to 6 months
- ○ Up to 1 year
- ○ Other (specify)

______

* 13. Taking into account the previous classification of surgical priorities (previous question), how long do you think it is safe to postpone **Priority 2** surgeries without causing harm to patient (make a general estimate of surgeries of this class of priority)

- Up to 1 month
- Up to 2 months
- Up to 3 months
- Up to 6 months
- Up to 1 year
- Other (specify)

* 14. Taking into account the previous classification of surgical priorities (previous question), how long do you think it is safe to postpone **Priority 3** surgeries without causing harm to patient (make a general estimate of surgeries of this class of priority)

- Up to 1 month
- Up to 2 months
- Up to 3 months
- Up to 6 months
- Up to 1 year
- Other (specify)

* 15. In relation to your income (compared to the previous 3 months before COVID-19 epidemics), in the last 4 weeks (since the beginning of the pandemic):

- Remained stable
- Reduced up to 25%
- Reduced 25-50%
- Reduced 50-75%
- Reduced more than 75%
- Increased more than 10%
- Other (specify)

* 16. In the past 4 weeks, which percentage (%) of UROLOGIC consultations have you performed in each of the following modalities? (the total must be **100%**):

Medical face-to-face consultation (in clinics or ambulatories) ____

Video-consultation (computer or cell phone camera) ____

Voice consultation, by phone or internet ____

* 17. In the last 4 weeks, if you have performed video-consultations, how frequently did you charge medical bills?

- ○ I charged medical bills (private of medical company) in more than 80% of video-consultations
- ○ I charged medical bills (private of medical company) in 50%-80% of video-consultations
- ○ I charged medical bills (private of medical company) in less than 50% of video-consultations
- ○ I did not perform video-consultations

* 18. In the past 4 weeks, after proposing video-consultations, how was the reaction/acceptance of patients:

- ○ More than 80% agreed with video-consultation
- ○ 50-80% of patients agreed with video-consultation Less
- ○ than 50% of patients agreed with video-consultation
- ○ I did not propose video-consultations

* 19. In the past 4 weeks, when you performed video-consultations, how did you feel in relation to missing some relevant diagnosis or treating correctly the patient due to the limitations of this kind of consultation?

- ○ I felt secure in more than 80% of consultations
- ○ I felt secure in 50-80% of consultations
- ○ I felt secure in less than 50% of consultations
- ○ I did not perform video-consultations

4. Professional History - Continuation

* 20. In the past 4 weeks, the perspective of income reduction in the next months made you reduce which of the following expenses in your professional practice? (more than one answer is allowed)

- [ ] None, for now
- [ ] I dismissed one or more employees
- [ ] I reduced investment in marketing/divulgation (social media, website, Google, other) Devolvi
- [ ] I returned my rented office
- [ ] I closed my office
- [ ] Other (specify)

* 21. In the past 4 weeks, the perspective of income reduction in the next months made you reduce which of the following expenses in your PERSONAL LIFE? (including wife and kids expenses) (more than one answer is allowed)

- [ ] None, for now
- [ ] I reduced schooling expenses (I moved my son to a cheaper school, I suspended private lessons/preparatory courses)
- [ ] I reduced my medical health plan (changed to a cheaper one or completely suspended)
- [ ] I reduced supermarket expenses
- [ ] I dismissed maid and/or cleaner
- [ ] I reduced costs with telephone/cell phones, internet, cable TV
- [ ] I reduced expenses with health professionals (therapist, dentist, etc)
- [ ] I reduced expenses with gym and/or personal trainer
- [ ] I suspended the rent of vacation home (beach, field)
- [ ] I sold car, motorcycle, boat or other not needed (that had costs)
- [ ] I suspended music, dance, theater or other classes
- [ ] I suspended foreign language courses
- [ ] Other (specify)

* 22. If your income reduction due to COVID-19 continues at least until SEPTEMBER, which of the following expenses do you think will have to reduce in your professional practice (more than one answer is allowed):

- [ ] None
- [ ] Dismiss one or more employees
- [ ] Reduce investment in divulgation/marketing (social media, website, Google, etc)
- [ ] I will give up attending a scheduled Medical congress
- [ ] I will suspend (rental) my office
- [ ] I will close my office
- [ ] Other (specify)

* 23. If your income reduction due to COVID-19 continues at least until SEPTEMBER, which of the following expenses do you think will have to reduce in your personal life (including partner and kids (more than one answer is allowed):

- [ ] None
- [ ] I will reduce health care plan cost (change to a cheaper one or suspend)
- [ ] I will reduce supermarket expenses
- [ ] I will dismiss maid/cleaner
- [ ] I will reduce costs of telephone/cell phone, internet, cable TV
- [ ] Reduce spending on healthcare professionals (therapy, dentist, etc.)
- [ ] I will suspend leisure trip in the second semester or summer vacation
- [ ] I will reduce gym/personal trainer expenses
- [ ] I will cancel lease of summer house (beach, field)
- [ ] I will sell car, motorcycle, boat or other unnecessary assets with costs
- [ ] I will suspend music, dance, theater or similar classes
- [ ] I will suspend foreign language classes
- [ ] Other (specify)

* 24. How physically healthy are you at present?

- ( ) Very healthy
- ( ) Moderately healthy
- ( ) Poorly healthy

* 25. Which is your current weight (kilograms)?

50 100 150+

* 26. Which is your height (centimeters)?

160 cm 190+ cm

* 27. In the past 4 weeks (since intensification of COVID 19 epidemics), YOUR WEIGHT:
:

- Slightly reduced (loss of 2 to 3 kilograms) Significantly
- reduced (loss of more than 3 kilograms)
- Remained stable
- Other (specify)
- Slightly increased (gain of 2-3 kilograms)
- Significantly increased (gain of more than 3 kilograms)
- I do not know

* 28. In relation to PHYSICAL ACTIVITY frequency in the past 4 weeks (since intensification of COVID-19 pandemic):

- Physical activity less frequent
- Same frequency of physical activity
- More frequent physical activity
- Other (specify)

* 29. Which physical activity have you been more frequently practiced in the past 4 weeks of confinement due to COVID-19?

- ○ Walking
- ○ Running
- ○ Exercises (abdominal, push ups and squats and others)
- ○ Weight training
- ○ Tennis
- ○ Yoga
- ○ Swimming
- ○ Pilates
- ○ Other (specify)

[ ]

5. Personal History

* 30. In relation to ALCOHOL BEVERAGE CONSUMPTION in the last 4 weeks (since intensification of COVID-19) you:

- Reduced consumption
- Consumption remained stable
- Increased consumption
- I don't drink alcoholic beverages

* 31. In relation to SEXUAL ACTIVITY (sexual relations) in the past 4 weeks (since intensification of COVID 19) you have had:

- More frequent sexual relations
- Same frequency of sexual relations
- Less frequent sexual relations
- I am sexually inactive for months/years

* 32. In relation to masturbation in the past 4 weeks (since intensification of COVID-19) you:

- Masturbate more frequently
- Same frequent masturbation
- Less frequent masturbation
- I do not masturbate for months/years

* 33. Please number the activities that you have been attended off duty in order of frequency (1 HIGHER FREQUENCY and 13 LOWER FREQUENCY)

- [ ] Social media (Facebook, Instagran, whatsapp groups)
- [ ] Household chores (kitchen, cleaning, gardening)
- [ ] Physical activities

TV or internet films, series, or other entertainment programs

TV news

Internet searches (news, shops, sports, culture)

Urology studies (revisions, new articles, books, academic papers)

Study of medical aspects of coronavirus

Non-medical books

Spiritual/religion activities

Expending time with Family (play, games, film sessions)

Play a musical instrument

Others

* 34. How worried are you with the possibility to work at the front line to consult contaminated COVID 19 patients?

- ◯ Very worried
- ◯ Moderately worried
- ◯ Little worried
- ◯ Not worried at all

* 35. Which would be your reaction if your employer (public or private) decide to allocate you to consult patients contaminated by COVID-19?

- ◯ I do not have an employer so I am not at risk
- ◯ I work at an Emergency Room and I am already consulting patients with coronavirus infection
- ◯ I am voluntarily working with contaminated patients with COVID 19
- ◯ I would accept, even if the protection conditions were not ideal
- ◯ I would accept only if protection conditions were ideal
- ◯ I would not accept even with correct protection
- ◯ Other (specifiy)

[ ]

* 36. Have you had symptomatic infection by coronavirus?

- ◯ Yes, with unequivocal confirmation (laboratory, tomography, other)
- ◯ Probably yes (clinical and epidemiologic suspected symptoms, but without confirmation)
- ◯ Very unlikely (clinical and epidemiologic symptoms less suspected)
- ◯ No

* 37. In case of confirmed symptomatic infection by coronavirus, which was the severity of the disease:

- ◯ I did not have
- ◯ Mild presentation
- ◯ Mild symptoms, without shortness of breath, without hospitalization
- ◯ Other (specify)

[ ]

- ◯ Severe symptoms, with shortness of breath, need of hospitalization, but no intubation
- ◯ Severe symptoms, with shortness of breath, need of hospitalization and intubation

* 38. Using a scale from 0 to 10, being 0 the less possible chance and 10 the higher possible chance, which number would you use to classify the possibility that in the beginning of SEPTEMBER 2020 we will have overcome most part of COVID-19 economic effects and in your medical practice?

0 lower chance | 5 | 10 higher chance

* 39. Which measures should Brazilian Society of Urology propose to improve urologist situation during COVID-19 pandemic?
